# Identification of genetic loci conferring seed coat color based on a high-density map in soybean

**DOI:** 10.3389/fpls.2022.968618

**Published:** 2022-08-01

**Authors:** Baoqi Yuan, Cuiping Yuan, Yumin Wang, Xiaodong Liu, Guangxun Qi, Yingnan Wang, Lingchao Dong, Hongkun Zhao, Yuqiu Li, Yingshan Dong

**Affiliations:** ^1^College of Agronomy, Jilin Agricultural University, Changchun, China; ^2^Soybean Research Institute, Jilin Academy of Agricultural Sciences, National Engineering Research Center for Soybean, Changchun, China; ^3^Crop Germplasm Institute, Jilin Academy of Agricultural Sciences, Changchun, China

**Keywords:** soybean, re-sequencing, high-density genetic map, seed coat color, QTL

## Abstract

Seed coat color is a typical evolutionary trait. Identification of the genetic loci that control seed coat color during the domestication of wild soybean could clarify the genetic variations between cultivated and wild soybean. We used 276 F_10_ recombinant inbred lines (RILs) from the cross between a cultivated soybean (JY47) and a wild soybean (ZYD00321) as the materials to identify the quantitative trait loci (QTLs) for seed coat color. We constructed a high-density genetic map using re-sequencing technology. The average distance between adjacent markers was 0.31 cM on this map, comprising 9,083 bin markers. We identified two stable QTLs (*qSC08* and *qSC11*) for seed coat color using this map, which, respectively, explained 21.933 and 26.934% of the phenotypic variation. Two candidate genes (*CHS3C* and *CHS4A*) in *qSC08* were identified according to the parental re-sequencing data and gene function annotations. Five genes (*LOC100786658*, *LOC100801691*, *LOC100806824*, *LOC100795475*, and *LOC100787559*) were predicted in the novel QTL *qSC11*, which, according to gene function annotations, might control seed coat color. This result could facilitate the identification of beneficial genes from wild soybean and provide useful information to clarify the genetic variations for seed coat color in cultivated and wild soybean.

## Introduction

Cultivated soybeans [*Glycine max* (L.) Merr.] were domesticated from wild soybeans by long-term targeted selection and improvement (Schmutz et al., 2010; Kim et al., 2012). The process of crop domestication encompasses a broad range of phenotypic changes throughout the multiple and continuous transition stages (Meyer and Purugganan, 2013). To better clarify the genetic mechanisms of this process, many scientists have researched the whole-genome information of wild and cultivated soybean genomes to obtain a clearer picture of the modes of soybean domestication and diversification (Lam et al., 2010; Li et al., 2011; Li Y. H. et al., 2014; Zhou et al., 2015; Han et al., 2016; Sedivy et al., 2017; Liu et al., 2020). Individually, they assembled *de novo* different wild and cultivated soybean genomes and constructed a graph-based genome to reveal numerous genetic variations and gene fusion events. This novel information enables the search for candidate genes that have played important roles in soybean domestication and improvement.

Cultivated soybeans have a lower genetic diversity after domestication than their wild counterparts. The lower diversity has potentially resulted in the loss of genes that might be important in different environments (Hyten et al., 2006; Qi et al., 2014a). Therefore, wild soybeans that exhibit high allelic diversity may be an important resource for reintroduction into domesticated genes. The populations constructed by crossing cultivated and wild soybeans were more conducive to the identification of beneficial genes associated with the soybean domestication process.

Seed coat color is a typical domestication trait, evolving from black to yellow, green, brown and double color during soybean domestication from wild to cultivated (Han et al., 2016; Liu et al., 2021). Soybean seed coat color is mainly controlled by five genetic loci, designated as *I*, *R*, *T*, *W1*, and *O* classical genetic loci in previous reports (Senda et al., 2002a). The loci *I*, *R*, and *T* regulated seed coat color by controlling the synthesis of seed coat pigments (Song et al., 2016). In addition, Guiamet identified the cytoplasmic genetic locus *CytG* in plant chloroplasts (Guiamét et al., 2002). With the development of molecular biotechnology, more than 30 molecular marker loci on different chromosomes that control seed coat color in soybean have been detected. Researchers tended to construct the genetic map by mapping a population to provide an essential framework for the putative quantitative trait loci (QTLs) and genes (Githiri et al., 2007; Ohnishi et al., 2011; Qi et al., 2014b). Song et al. (2016) used a biparental population developed from the cross between two cultivated soybeans with yellow seed color and brown seed color to confirm the locus and in which different seed coat colors were further dissected into simple trait pairs. By genotyping the entire F_2_ population using flanking markers located in fine-mapping regions, the genetic basis of seed coat color was dissected. Du et al. (2019) constructed a high-density linkage map of the recombinant inbred lines (RILs) population by using a specific length amplified fragment (SLAF) technique and determined the QTL of seed coat color and seed size for sesame. Zhang et al. (2019) used the RIL population derived from crossing 09A001 to identify the major and minor QTLs controlling seed coat color in *Brassica rapa* L. Li et al. (2021) identified the candidate genes regulating seed coat color in sesame using QTL mapping and transcriptome analysis by F_2_ populations. Liu et al. (2021) used an improve wild soybean chromosome segment substitution line (CSSL) population from NN1138-2(*max*) × N24852(*soja*) to identify wild vs. cultivated gene alleles conferring seed coat color and days to flowering in soybean. They identified the same trait in different populations to identify consistent QTLs (Oyoo et al., 2011). A total of 20 genes were reported, and 15 of them were in the flavonoid metabolic pathway. The accumulation of flavonoid substances in dynamic equilibrium was the result of the interaction of transcription factors (Gillman et al., 2011; Cho et al., 2019; Jia et al., 2020). In addition, the interaction of some MYB (v-myb avian myeloblastosis viral oncogene homolog) transcription factors regulate the accumulation of flavonoid substances (Albert et al., 2014). Transcription factors such as GmMYB39 and GmMYB100 could negatively regulate the synthesis of isoflavones in soybean hairy roots (Liu et al., 2013; Yan et al., 2015). GmMYB58, GmMYB176, and GmMYB205 could positively regulate the synthesis of isoflavones (Yi et al., 2010; Han et al., 2017). The MYB transcription factors GmMYBA2 and GmMYBR are identified as transcriptional activators in a feedback loop to control the pigmentation of seed coat in soybeans (Gao et al., 2021). However, the genetic information controlling seed coat color during soybean domestication has not been completely elucidated and the transcriptional regulation relationship among the loci remains elusive.

To identify the loci and genes that controlling seed coat color, we used 276 F_10_ RIL populations developed from a cross between *Glycine max* and *Glycine soja* as the materials to construct a high-density genetic map by whole genome re-sequencing, map the additive QTLs, and predict candidate genes for seed coat color. The results of this study could facilitate the identification of beneficial genes from wild soybean and lead to a greater understanding of the process of soybean domestication.

## Materials and methods

### Plant materials and DNA extraction

The F_10_ RIL population (*n* = 276) was developed from a cross between Jiyu47 (JY47) and ZYD00321 using a single seed descent method. JY47 is an outstanding cultivated soybean with a yellow seed coat, ZYD00321 is a typical wild soybean with a black seed coat. The two parents and the RIL populations were planted in pots in the Gongzhuling Experiment Station at the Jilin Academy of Agricultural Sciences. We employed a planting pattern of two seeds per pot in three replicates to preserve the uniform density.

Fresh leaf tissue from the two parents and RIL individuals was collected at the flowering stage, immediately frozen in liquid nitrogen, then stored in a −80°C freezer. To obtain the high-quality DNA, the cetyltrimethylammonium bromide (CTAB) method was used to extract genomic DNA (Zhang et al., 2005). The quality and concentration of the total genomic DNA were spectrophotometrically assessed by the optical density value (OD_600_ = 230/260, 260/280). The sequencing libraries were constructed following the manufacturer’s instructions.

### Genome re-sequencing and high-density genetic map construction

We performed whole-genome re-sequencing on RIL populations and the two parents to construct our high-density genetic map. Genome re-sequencing was constructed on the Illumina HiSeq2500 platform. We used an average sequencing depth of 20.00-fold in the two parents and 3.00-fold for individual RILs, and compared the sequence data with *Williams 82* (Glycine_max_v2.1) reference genome using the BWA package (Li and Durbin, 2009a) and combined the co-segregating markers which had been produced by the GATK process after comparison into bins using the HighMap software (Li et al., 2009b).

The HighMap software was used to analyze the linear arrangement of the bin markers within 20 linkage groups (LGs) and estimate the genetic distance between adjacent markers (Liu et al., 2014). The polymorphic single nucleotide polymorphisms (SNPs) were aligned with the reference genome and mapped onto 20 chromosomes (Chr). We calculated the MLOD scores between the polymorphic markers and filtered for MLOD values of less than 5. The HighMap software was used to calculate the map distances. SMOOTH (Van Ooijen, 2006) was applied to correct errors based on the parental contribution of the genotypes and a k-nearest neighbor algorithm was applied to impute missing genotypes. We mapped skewed markers by applying a multipoint method of maximum likelihood and estimated the map distances using the Kosambi mapping function in centimorgan (cm).

### Phenotypic evaluation

We followed the “*Descriptors and Data Standard for soybean* (*Glycine* spp.)” (Qiu et al., 2006) to classify the traits and used the numbers 1–5 to represent the yellow, green, black, brown, and double color, respectively. The identified phenotypic data were collected and analyzed. We used Excel 2019 for statistics on all the phenotypic data and the software Graphpad prism 8.0 (Swift, 1997) for graphing.

### Quantitative trait loci mapping and candidate genes prediction

The composite interval mapping (CIM) method of the *R/qtl* package (Arends et al., 2010) was used to detect additive QTLs for seed coat color. A total of 1,000 permutation tests at the 95% confidence level were used to set the logarithm-of-odds (LOD) threshold to detect significant QTLs (Churchill and Doerge, 1994). Based on 1,000 permutations, LOD = 5.356 was used to determine the presence of a putative QTL associated with the target trait in a particular genomic region. The QTLs were named as per the guidelines described (Swift, 1997). The sequences within the target QTLs were analyzed according to the *Williams 82* soybean reference genome sequence (Glycine_max_v2.1) in National Center for Biotechnology Information (NCBI). The physical positions of target intervals were aligned based on the same reference genome sequence. We obtained the SNPs and insertion-deletion (InDels) in the target intervals from the re-sequencing data and the genes with sequence variations between two parents to predict the candidate genes. We arranged the distributions of SNPs or InDels upstream, in the intragenic region and downstream.

We used the BLAST search on Soybase^1^ to search for descriptions of the soybean genes. The CDS sequences from the QTL regions were retrieved from Phytozome^2^. The putative functions of the candidate genes were annotated based on the gene ontology (GO)^3^ and Kyoto Encyclopedia of Genes and Genomes (KEGG)^4^ databases. We listed genes with similar functions or functional domains as the major candidate genes according to gene annotations and the functional analysis.

## Results

### Population sequencing and high-density genetic map construction

Recombinant inbred line populations derived from a cross between JY47 and ZYD00321 were sequenced on the Illumina HiSeq2500 platform to construct a high-density genetic map. A total of 20.85 Gb of clean data was obtained for JY47 and 20.99 Gb for ZYD00321 with 20.0-fold and 21.0-fold depth, respectively. The sequencing quality values (Q30) of the two parents were >93.00% and the GC content percentages (the proportion of Guanine and Cytosine of the whole genome) were, respectively, 35.76 and 35.84% (Supplementary Table 1).

**FIGURE 1:**
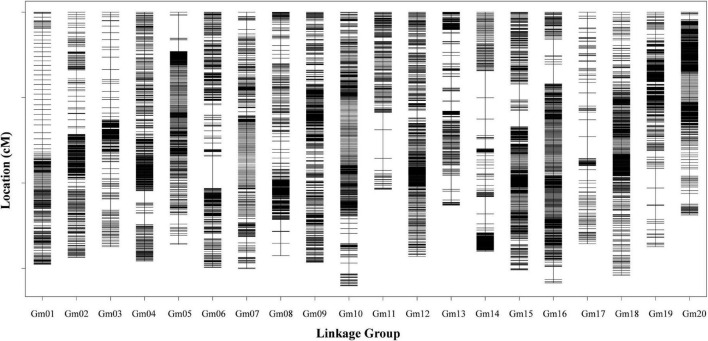
The soybean high-density genetic map. Bin markers are distributed on 20 chromosomes. The black bars in each linkage group represent the mapped bin markers. The linkage group number is shown on the *x*-axis and genetic distance is shown on the *y*-axis (cM is the unit).

A total of 2,612,708 SNPs between the parents were identified using the BWA package by comparing the sequencing data to the Williams 82 reference genome. The alignment efficiency was 96.26%. We obtained a total of 854.08 Gb of clean data with approximately 3.09-fold depth for each RIL. The average Q30 for the sequencing was 93.17% and the average GC content was 35.98% for each RIL. After filtering and quality assessment, 9083 bin markers without recombination events were used to construct the genetic map (Figure 1). The genotype of the RIL populations was generated to evaluate the genetic map quality. We used different colors to represent the origin of the different DNA fragments according to the physical location of 9,083 bin markers on 20 chromosomes (Supplementary Figure 1). It showed that this RIL population with a high recombination frequency was suitable for genetic analysis using marker-density linkage maps. A high-density genetic map with a total length of 2814.07 cM was constructed and the average distance between adjacent markers was 0.31 cM (Table 1). The genetic length of 20 LGs ranged from 103.69 cM (Chr11) to 160.19 cM (Chr10). The largest average distance was 1.01 cM on Chr17 with 135 bin markers and the smallest average density was 0.20 cM on Chr15 with 739 bin markers. The largest gap was mapped to Chr06 and was 18.82 cM in length. The proportion of gaps <5 cM between two markers was 94.33%.

**TABLE 1:** Characteristics of the high-density genetic map.

Linkage group ID	Total marker	Total distance (cM)	Average distance (cM)	Max gap (cM)	Gaps < 5 cM (%)
Chr01	389	147.54	0.38	4.63	94.33%
Chr02	472	143.55	0.30	5.22	99.58%
Chr03	264	137.34	0.52	7.20	98.86%
Chr04	590	145.77	0.25	7.07	99.66%
Chr05	514	135.98	0.27	13.47	99.42%
Chr06	448	149.49	0.33	18.82	98.43%
Chr07	345	150.19	0.44	5.44	99.71%
Chr08	372	142.60	0.38	7.31	99.19%
Chr09	645	146.43	0.23	3.08	100.00%
Chr10	665	160.19	0.24	6.36	99.40%
Chr11	219	103.69	0.48	16.78	98.62%
Chr12	604	143.01	0.24	4.56	100.00%
Chr13	267	113.04	0.42	8.30	96.24%
Chr14	335	140.00	0.42	15.76	98.20%
Chr15	739	150.91	0.20	9.34	99.73%
Chr16	499	158.52	0.32	11.53	99.40%
Chr17	135	135.57	1.01	12.86	95.52%
Chr18	596	154.16	0.26	3.26	100.00%
Chr19	448	137.35	0.31	11.38	99.33%
Chr20	537	118.73	0.22	6.24	98.88%
Total	9083	2814.07	0.31	18.82	94.33%

To evaluate the collinearity between the genetic map and the soybean reference genome, 9083 bin markers were mapped to the soybean reference genome. A collinearity analysis showed that the order of markers on 20 chromosomes was consistent with the genome (Supplementary Figure 2). Consecutive curves between physical distances and genetic distances were observed except on Chr11 and Chr14. The Spearman coefficients of 20 LGs were >0.99 and collinearity was high at 99.80%, which indicated that the genetic and physical positions followed an identical order on this map. The high collinearity on our map indicated the genetic recombination rate was accurate and the gene annotation within QTL intervals was reliable.

### Phenotypic variation of seed coat color

**FIGURE 2:**
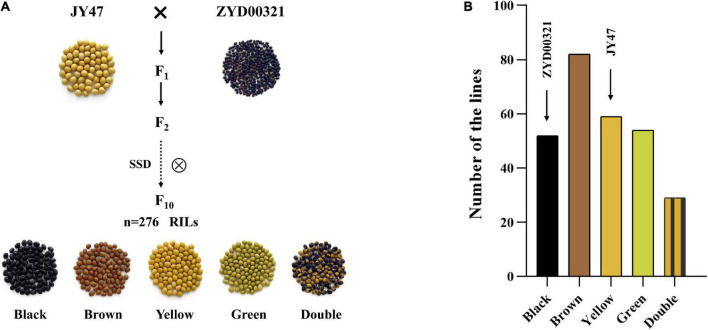
Phenotypic identification of parents and RIL populations. **(A)** The characteristics of the seed coat color of JY47 and ZYD00321 and RIL populations. **(B)** Frequency distribution of seed coat color for the 276 RILs.

Seed coat color is a qualitative trait that is difficult for the environment to affect and had reached a state of purity for the F_10_ RIL population. Great phenotypic variation existed among the 276 RILs (Figure 2A). Five phenotypic types were found in the RIL population: black, brown, yellow, green, and double color (Figure 2B). The frequency distribution indicated that this RIL population was isolated for this trait and fulfilled the essential conditions for QTL localization. Of the 276 RILs, the brown seed coat was present in the largest quantity and the double color in the least quantity in the five classifications (Supplementary Table 2).

### Quantitative trait loci mapping for seed coat color

**FIGURE 3:**
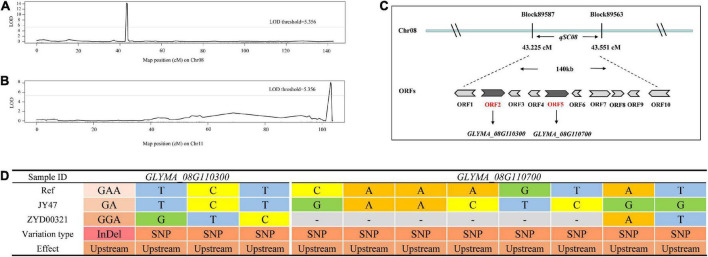
Fine mapping of two QTLs. **(A)** Mapping of QTLs for seed coat color on Chr08. The curves indicate the physical position of markers against the LOD score of the QTLs detected on Chr08. **(B)** Mapping of the QTLs for seed coat color on Chr11. The curves indicate the physical position of the markers against the LOD score of the QTLs detected on Chr11. **(C)** Distribution of tightly linked markers and plausible candidate genes for seed coat color on *qSC08* in soybean. **(D)** SNP/InDels variants based on re-sequencing data from both parents of *GLYMA_08G110300* and *GLYMA_08G110700*.

Based on the constructed high-density genetic linkage map and the identified phenotypic analysis of seed coat color, we used the *R/qtl* package and the CIM program to identify QTLs associated with seed coat color in the RIL population (*n* = 276). The threshold of LOD scores for estimating the significant QTL effects was determined using 1,000 permutations. In total, two QTLs related to seed coat color designated as *qSC08* and *qSC11* were detected on Chr08 and Chr11, respectively (Table 2). The LOD score curves were constructed and sharp peaks spanning Chr08 and Chr11 were obtained (Figures 3A,B). The high phenotypic variance, respectively, explained by two QTLs ranged from 21.933 to 26.934% and the LOD score was 8.112 and 14.251. The additive effects of *qSC08* and *qSC11* were, respectively, −0.616 and −0.683 and the beneficial alleles of two major and stable QTLs were derived mainly from the male parent ZYD00321 (Table 2). The results indicated that two loci *qSC08* and *qSC11* had a powerful effect on the seed coat color.

**TABLE 2:** Two QTLs for seed coat color in RIL populations.

Name	Chr	Genetic interval	Physical interval	Marker interval	LOD	ADD	PVE (%)
*qSC08*	D1b	43.225–43.551	8449385–8588340	Block89587–Block89563	14.251	−0.616	21.933
*qSC11*	F	102.961–103.143	11236206–22112949	Block125748–Block126245	8.112	−0.683	26.934

### Gene annotation and candidate genes prediction

**FIGURE 4:**
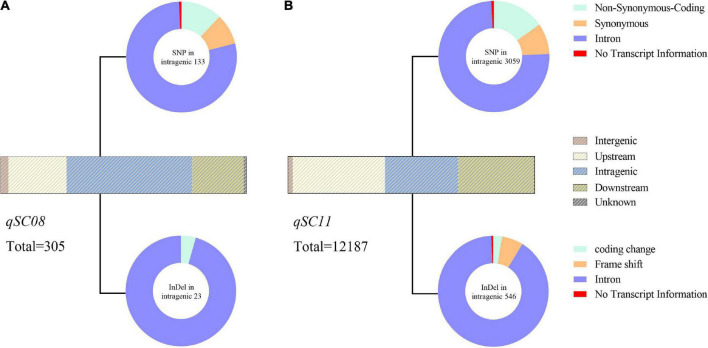
Analysis of SNPs and InDels between two parents in *qSC08* **(A)** and *qSC11* **(B)**. Strip-shape charts show the distribution of SNPs and InDels in different genomic regions. The upstream and downstream represent the 5 kb within the region of transcription start and stop sites, respectively. Pie charts show the effects of SNP (upside) and InDel (underside) in the intragenic regions. And the corresponding quantity of SNP or InDel is labeled near the pie chart.

To validate the QTL mapping results, we annotated and analyzed the potential genes within the QTL intervals by comparing the genome interval regions within the QTLs with the reference genome sequences. The 0.326 cM physical interval for *qSC08* represents approximately 140 kb in the reference genome and contains 10 candidate genes according to the annotation of *Williams 82* (Figure 3C). We analyzed the SNPs and InDels based on the whole genome re-sequencing data of both parents (JY47 and ZYD00321) to understand the genetic variations of these genes. In *qSC08*, 8 of 10 genes possessed SNPs or InDels. In total, 254 SNPs and 51 InDels were detected among 8 genes (Supplementary Table 3). Among these variations, a percentage of 44.59% (136/305) were located outside of the genes, including the scope within or beyond 5 kb upstream and downstream of the transcription start and stop sites. A percentage of 44.59% variations were located in the intergenic region. Non-synonymous variations with a percentage of 9.83% (30/305) were found in the coding sequence among the intragenic region (Figure 4A and Supplementary Table 3). Additionally, we annotated the functions of 8 variant genes based on the GO and KEGG databases to anchor the candidate genes for seed coat color in soybean (Supplementary Table 4). The results indicated that *LOC100789075* (*GLYMA_08G110300)* and *LOC100779649* (*GLYMA_08G110700)* might be involved in the response to seed coat color in soybean. They encoded chalcone synthase (*CHS3C* and *CHS4A*) and were involved in the flavonoid biosynthetic pathway. Chalcone synthase (*CHS*) is a key enzyme in the branch of the phenylpropanoid pathway leading to the biosynthesis of flavonoid pigments including anthocyanins. A sequence comparison analysis between the parents supported the above prediction. SNP or InDel variations between both parents were found in the upstream regions of the two genes; one InDel and three SNPs for *GLYMA_08G110300* and eight SNPs for *GLYMA_08G110700* (Figure 3D). Based on the functional annotation of candidate genes and sequence alignment analysis between the two parents, we predicted *GLYMA_08G110300* and *GLYMA_08G110700* as candidate genes that controlled seed coat color in soybean.

Based on the Williams 82 soybean reference genome, a total of 281 genes occupied the novel *qSC11* were identified. We analyzed the SNPs/InDels based on the whole genome re-sequencing data of two parents to understand the genetic variations of these genes. A total of 256 variant genes contained 9,996 SNPs and 2,191 InDels in *qSC11* were identified as the candidate genes for seed coat color (Supplementary Table 5). Among these variations, a substantial portion (68.48%) was located outside of the genes, including the scope within or beyond 5 kb upstream and downstream of the transcription start and stop sited (Figure 4B). Only 29.58% of variations were located in the intragenic region (Figure 4A and Supplementary Table 5). Non-synonymous variations with a percentage of 15.27% (467/3059) were found in the coding sequence among the intragenic region (Figure 4B and Supplementary Table 5). It was speculated that the key genes regulating seed coat color existed in the target intervals from the mass of genetic variations between the parents.

**FIGURE 5:**
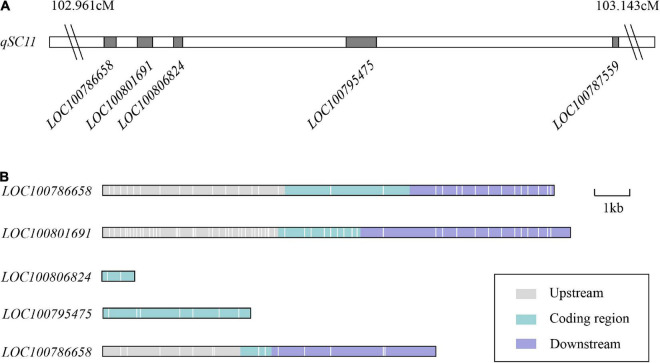
Distribution of the five candidate genes for seed coat color on *qSC11*. **(A)** Distribution of the candidate genes on *qSC11*. **(B)** Variation distribution of the candidate genes on *qSC11*. The upstream and downstream represent the 5 kb within the region of transcription start and stop sites, respectively. The regions are markers of different colors. Each white line represents one SNP/InDel variant.

The gene functions of 256 variant genes were annotated to anchor the candidate genes for soybean seed coat color based on GO and KEGG databases, among which, only 131 genes were annotated (Supplementary Table 6). A total of 122 genes were annotated in the GO database as cellular components, molecular functions and biological processes (Supplementary Figure 3 and Supplementary Table 6), and 71 genes were detected in the KEGG database (Supplementary Table 6). According to the gene annotation results, five genes were annotated that might involve the biosynthetic pathway that controlled seed coat color, including *LOC100786658*, *LOC100801691*, *LOC100806824*, *LOC100795475*, and *LOC100787559* (Figure 5A and Supplementary Table 6). Of these, *LOC100786658* and *LOC100801691* encoded xanthoxin dehydrogenase and are involved in carotenoid biosynthesis. *LOC100806824* and *LOC100795475* encoded photosystem I reaction center subunit VI and protein TIC110 from chloroplast, respectively. *LOC100787559* encodes cytochrome P450. All the five genes existed with SNP or InDel variations in the coding region; *LOC100786658*, *LOC100801691*, and *LOC100787559* existed with SNP or InDel variations in the upstream, coding region and downstream between two parents; *LOC100806824* only existed two SNPs in the intragenic region; *LOC100795475* existed six SNPs and one InDels in the intragenic region, severally (Figure 5B and Supplementary Table 7). Among which, the candidate genes *LOC100795475* had two non-synonymous coding variations (Act/Gct, cGt/cAt) between two parents (Supplementary Table 7). All the five candidate genes were annotated as affecting the composition and content of pigments from seed coats in different ways.

## Discussion

### The high-density genetic map for quantitative trait loci mapping

A proper marker density for high-density genetic maps could provide an essential framework for QTL fine mapping (Gutierrez-Gonzalez et al., 2011; Qi et al., 2014a). In previous studies, the genetic maps constructed with restriction fragment length polymorphism (RFLP) and SSR markers have drawbacks of relatively few markers and large gaps, which limited the efficiency and accuracy of QTL mapping. With the completion of the whole genome sequencing of Williams 82 (the reference genome in this study) and the rapid development of sequencing technology, SNP markers have become widely used to construct genetic maps in plants (Hyten et al., 2008). Cai et al. (2018) used a high-density genetic linkage map containing 3,469 recombination bin markers based on 0.2 × RAD-seq technology to map QTLs for isoflavone content. Han et al. (2019) constructed a high-density genetic map using 260 RILs derived from the cultivars of Heihe43 and Heihe18, and the constructed map contained 4,953 SLAF markers spanning 1478.86 cM with an average distance between adjacent markers of 0.53 cM. Chu et al. (2021) reported on a genetic linkage map constructed by polymorphic 2,234 SNP markers from a SoySNP6K array, which covered a total of 4229.01 cM genetic distance with an average distance of 1.89 cM. Tian et al. (2022) constructed a high-density genetic map by re-sequencing technology, which contained a total of 4,011 recombination bin markers with an average distance of 0.78 cM in the entire RILs population. In this study, we used F_10_ RILs from the cross between JY47 and ZYD00321 to construct the high-density genetic map that contained 9,083 bin markers with an average distance of 0.31 cM between adjacent markers.

Although the resolution of genetic maps has been improved by increasing marker density, it has been limited by a Linkage disequilibrium (LD) in soybean that is significantly higher than in other plants (Lam et al., 2010; Gutierrez-Gonzalez et al., 2011). Because the average LD of cultivated soybean is approximately 150 kb, at least 6,300 distributed markers could theoretically fulfill a high-density genetic map (Li B. et al., 2014). In this study, we used the re-sequencing technology with high efficiency and capacity to construct a high-density genetic map. The number of bin markers was significantly higher than the theoretical value of the genetic map. Compared with the previously constructed high-density genetic maps, our map presented the characteristics of more markers (9083 bin markers), a smaller average genetic distance (0.31 cM) and higher collinearity (99.80%), which effectively eliminated the drawback of a large gap. These results indicated that the drawback of the high link disequilibrium could be avoided and fulfill the high-density genetic map could be achieved. Moreover, the use of RIL population with a wide range of variation can enhance our understanding of molecular mechanism evolution and genetic regulation, and also help to identify more QTLs regulating seed coat color in soybeans.

### Identification and evaluation of quantitative trait locis for seed coat color

High-generation RIL populations were excellent materials for QTL localization. In this study, we identified two major QTLs for seed coat color, *qSC08* and *qSC11*, by the F_10_ RIL population with significant isolation for seed coat color. However, the *qSC11* presented a much broader interval compared with *qSC08*, we speculate the reason was the non-uniform distribution of the 9,083 bin markers on 20 chromosomes. In comparison to the previous results, *qSC08* was mapped into a much smaller region (Ohnishi et al., 2011; Qi et al., 2014a; Liu et al., 2021). Senda et al. (2002b) and Song et al. (2016) had shown five classical genetic loci *I*, *R*, *T*, *W1*, and *O*. Coincidently, *qSC08* was located precisely within the classical genetic *I* locus that controlled seed coat color by regulating the distribution of anthocyanin and proanthocyanidin from seed coat (Todd and Vodkin, 1996). The *I* locus was located in the chalcone synthase *CHS* gene-rich region (Clough et al., 2004). The chalcone synthase gene family of soybean includes *CHS1*, *CHS2*, *CHS3*, *CHS4*, *CHS5*, *CHS6*, *CHS7*, *CHS8*, and *CHS9* (Clough et al., 2004). Notably, the candidate gene *CHS4* (*GLYMA_08G110700*) in our study is a member of the chalcone synthase gene family (Senda et al., 2002b). The clear conclusion was that the *qSC08* locus had a powerful effect on seed coat color in soybean and also demonstrated the accuracy and reliability of this study.

Three QTLs for seed coat color were detected on Chr11 in the previous studies. Of these, the classical genetic locus *K1* controlled the distribution of pigment in the saddle region, regulating seed coat color in soybean (Cho et al., 2017). The *D2* locus determines a yellow or green coat according to the chlorophyll content in the seed coat (Fang et al., 2014). Kovinich et al. (2011, 2012) detected a locus within the physical interval 1992156–1993544 on Chr11 and identified the candidate gene *Glyma.11g027700* that encoded anthocyanidin synthase *ANS3*. The previous reports had no QTL for seed coat color in the physical interval 11236206–22112949 on Chr11, so the target region *qSC11* was a novel QTL. We predicted five candidate genes from *qSC11*. Of these, *LOC100801691* and *LOC100786658* encoding xanthoxin dehydrogenase and are involved in carotenoid biosynthesis. Carotenoids are the second most abundant natural pigments with more than 750 members. The color of carotenoids varies from colorless to yellow, orange, and red with variations reflected in plants (Nisar et al., 2015). *LOC100806824* and *LOC100795475* encoded photosystem I reaction center subunit VI and protein TIC110 from the chloroplast. During photomorphogenesis, the chlorophyll and carotenoid compounds are promoted in a coordinated manner in the development of photosynthesis (Welsch et al., 2000; Rodríguez-Villalón et al., 2009). In chloroplasts, most carotenoid biosynthetic genes are activated during light-triggered de-etiolation (Giuliano et al., 2008; Rodríguez-Concepción, 2010), and it indirectly affecting the accumulation of pigments from the individual tissues of the plants. *LOC100787559* encodes cytochrome P450, which plays an important role in flavonoid biosynthesis and the principal cytochromes in plants (Tanaka, 2006; Severin et al., 2010; Waese et al., 2017). In the present study, SNP and InDel variations were also observed between the two parents in the genomic sequences of the five candidate genes, including the regions 5-kb downstream and upstream of the genes and the coding regions (Figure 5 and Supplementary Table 7). It was also found that the candidate genes *LOC100795475* occurred with non-synonymous coding variations (Act/Gct, cGt/cAt) between the two parents. Therefore, it was speculated that these genes might be the key genes responsible for soybean seed coat color. The cloning and functional analyses of these candidate genes will be conducted in the future, which will help to expound the genetic variations between wild and cultivated soybean more thoroughly regarding seed coat color.

### Identification of important loci and genes in wild soybean

Cultivated soybeans were domesticated from wild soybeans *via* long-term selection and improvement (Sedivy et al., 2017). In previous studies, researchers usually located and analyzed target traits by constructing populations. Cho et al. (2021) used a population derived from a cross between a Korean cultivar and IT162669 to identify QTLs conferring salt tolerance in soybean. Githiri et al. (2007) used an F_2_ population derived from a cross between two cultivated soybeans to identify five QTLs for pigmentation. Ohnishi et al. (2011) used two sets of RILs between two cultivars to identify minor QTLs for seed coat color. However, such procedures could not fully reflect the changes in seed coat color and might miss some vital genetic information during the domestication process.

Identification of genes and alleles from wild germplasm associated with seed coat color could allow deeper insight into the process of the changes in this trait during soybean domestication (Hyten et al., 2006; Lam et al., 2010; Zhuang et al., 2022). ZYD00321 is a typical wild soybean with a black seed coat, a small seed and a vining growth habit. We used the RIL population derived from JY47 (*Glycine max*) and ZYD00321 (*Glycine soja*) to identify QTLs and genes for seed coat color. Identification of the source of the beneficial alleles on each QTL is the prerequisite for the QTLs application to molecular breeding and crop improvement (Wang et al., 2007). In this study, *qSC08* and the novel interval *qSC11* showed consistent ADD (−0.616 and −0.683) and similar PVE (21.933 and 26.934%), which indicated that both beneficial alleles were derived from the wild soybean ZYD00321 and demonstrated that a wild soybean with a black seed coat had a crucial role in producing different seed coat colors, facilitating the identification of superior genes in the domestication process (Alkan et al., 2011; Li et al., 2011). The intact and accurate genomic information we obtained for wild germplasm was beneficial for identifying QTLs and conducting association studies on seed coat color (Kim et al., 2010; Xie et al., 2019).

## Data availability statement

The datasets presented in this study can be found in online repositories. The names of the repository/repositories and accession number(s) can be found below: https://www.ncbi.nlm.nih.gov/, PRJNA848661.

## Footnotes

1
https://www.soybase.org

2
https://phytozome-next.jgi.doe.gov/

3
https://www.ebi.ac.uk/QuickGO/

4
http://www.kegg.jp/
