# Supplementary material for: Identification of genetic loci conferring seed coat color based on a high-density map in soybean
Source: Front Plant Sci. 2022 Aug 1;13:968618. doi: 10.3389/fpls.2022.968618 (PMC9376438; doi:10.3389/fpls.2022.968618)
Supplement: Supplementary file 1 [file Data_Sheet_1.zip › Supplementary_Material.docx]

Supplementary Material

# Supplementary Figures


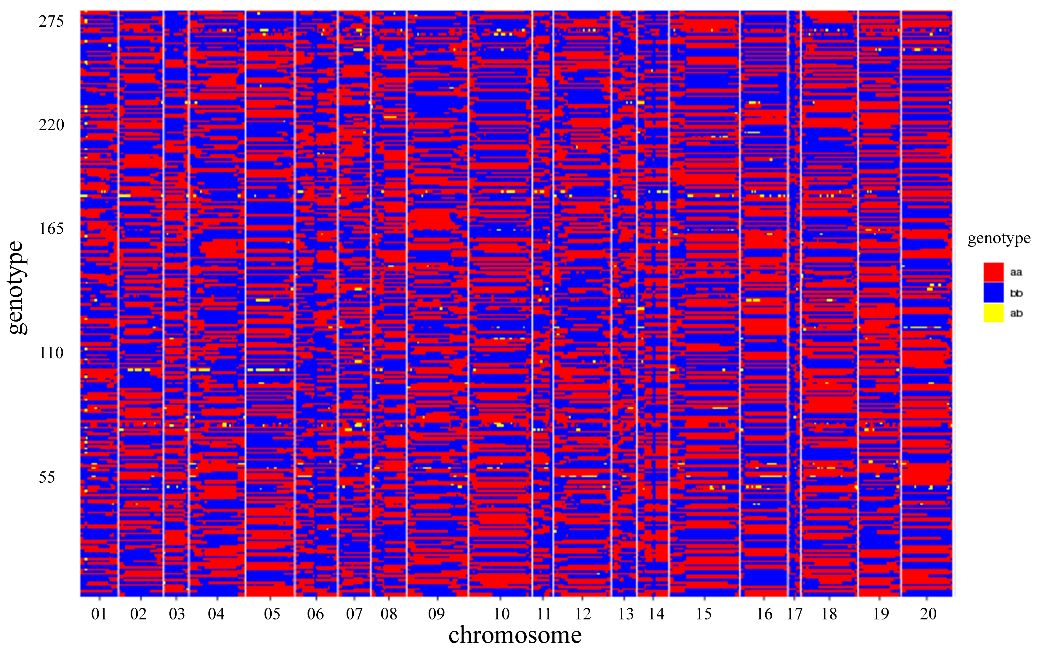


**Supplementary Figure 1.** The genotype of the RIL populations. The red regions show the genotype from ZYD00321, and the blue regions show the genotype from JY47. The yellow regions show heterozygous parts.

**
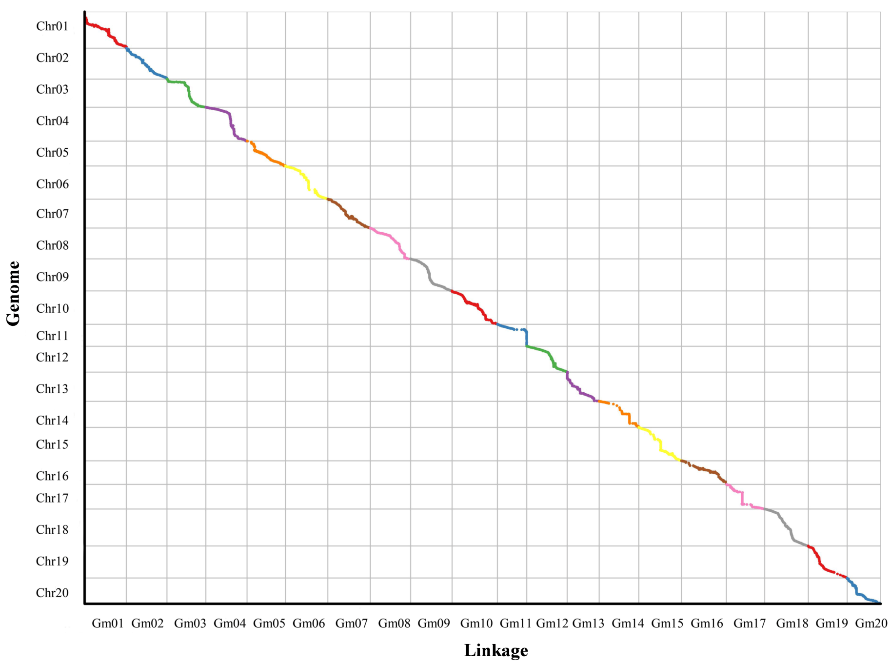
**

**Supplementary Figure 2.** Collinearity analysis of the genetic map and genome. The abscissa is the genetic distance of each linkage group, the ordinate is the physical length of each linkage group, which scatters the form of markers in the genome and genetic map collinearity. Different colors represent different chromosomes or linkage groups.


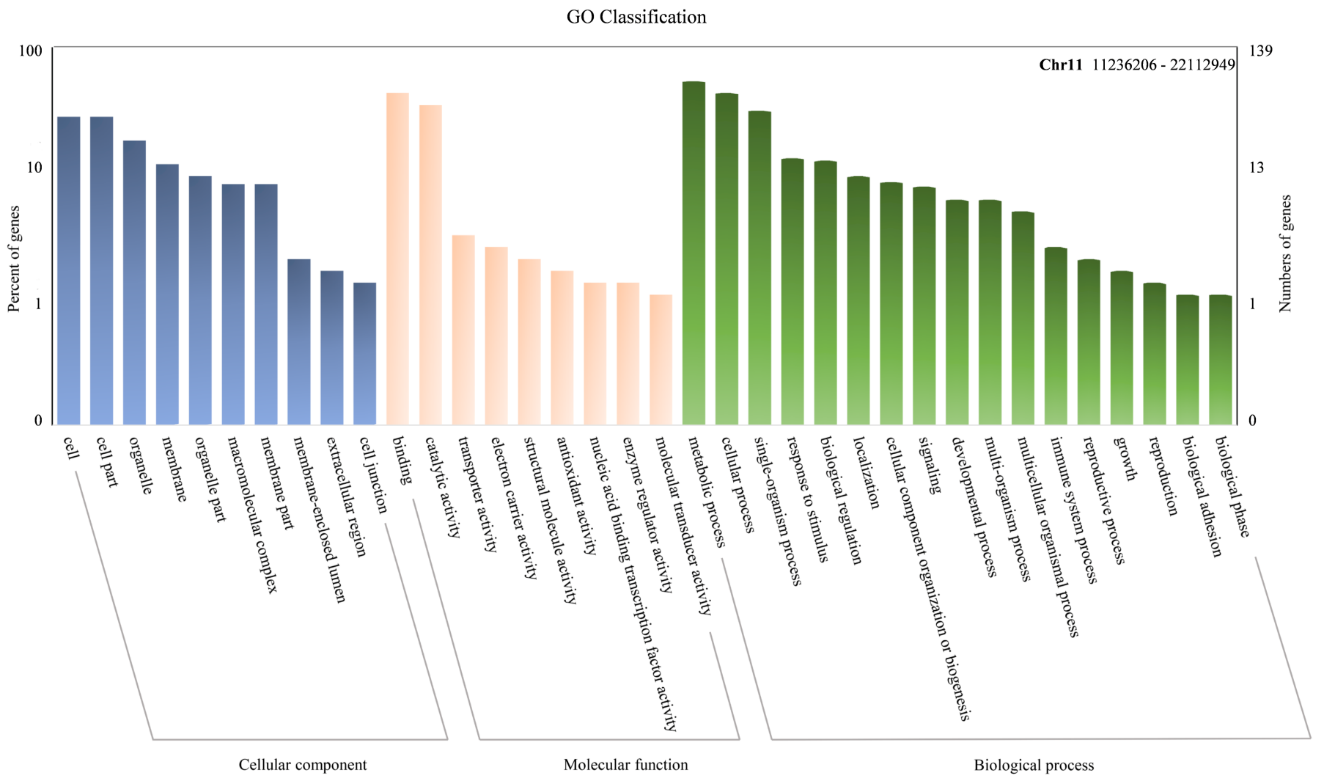


**Supplementary Figure 3.** Gene ontology (GO) annotation of genes within *qSC11*. The y axis on the right shows the number of genes in each category, and the y axis on the left shows the percentage of a specific category of genes in that main category.
